# Reactive Astrocytes with Reduced Function of Glutamate Transporters in the *App* ^NL‑G‑F^ Knock-in Mice

**DOI:** 10.1021/acschemneuro.4c00714

**Published:** 2025-05-27

**Authors:** Ipsit Srivastava, Julen Goikolea, Tamer Ayberk Kaya, María Latorre-Leal, Francesca Eroli, Marta Pereira Iglesias, Laura Álvarez-Jiménez, Luis Enrique Arroyo-García, Makoto Shimozawa, Per Nilsson, André Fisahn, Maria Lindskog, Silvia Maioli, Raúl Loera-Valencia

**Affiliations:** † Division of Neurogeriatrics, Center for Alzheimer Research, Department of Neurobiology, Care Sciences and Society, 27106 Karolinska Institutet, 17164 Solna, Sweden; ‡ Tecnologico de Monterrey, School of Medicine and Health Sciences, Campus Chihuahua, Av. H. Colegio Militar 4700, Nombre de Dios, 31150 Chihuahua, Chih. Mexico

**Keywords:** astrocytes, astrocytic glutamate transporters, GLT-1, GLAST, Alzheimer’s disease

## Abstract

Alzheimer’s disease (AD) is associated with synaptic and memory dysfunction. One of the hallmarks of AD is reactive astrogliosis, with reactive astrocytes surrounding amyloid plaques in the brain. Astrocytes have also been shown to be actively involved in disease progression, nevertheless, mechanistic information about their role in synaptic transmission during AD pathology is lacking. Astrocytes maintain synaptic transmission by taking up extracellular glutamate during synaptic activity through astrocytic glutamate transporter GLT-1, but its function has been difficult to measure in real-time in AD pathology. Here, we used an *App* knock-in AD model (*App* ^*NL‑G‑F*^) carrying the Swedish, Arctic and Beyreuther mutations associated with AD and exhibiting AD-like Aβ plaque deposition and memory impairment. Using immunohistochemistry, patch-clamp of astrocytes, and Western blot from tissue and FACS isolated synaptosomes, we found that *App* ^*NL‑G‑F*^ mice at 6–8 months of age have astrocytes with clearly altered morphology compared to wild-type (WT). Moreover, astrocyte glutamate clearance function in *App* ^*NL‑G‑F*^ mice, measured as electrophysiological recordings of glutamate transporter currents, was severely impaired compared to WT animals. The reduction of glutamate uptake by astrocytes cannot be explained by GLT-1 protein levels, which were unchanged in synaptosomes and hippocampus of *App* ^*NL‑G‑F*^ mice. Our data suggest that astrocytic glutamate transporters are affected by excess Aβ42 in the brain contributing to synaptic dysfunction in the hippocampus. This data contributes to the notion of restoring astrocyte synaptic function as a potential therapeutic strategy to treat AD.

## Graphical abstract

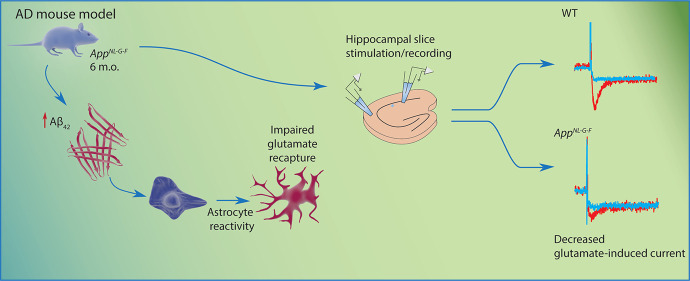

## Introduction

Alzheimer’s disease (AD) is a form of dementia characterized by a continuous decline in cognitive functions. It is the most prevalent form of dementia and is estimated to affect 20–40% of the population over 85 years.^1^ With more than 400 failed clinical trials in the search for a treatment for the disease, there is still no widely available cure or treatment to slow down AD progression. The most notable feature of AD is the accumulation of the amyloid β peptide (Aβ), hyperphosphorylated tau in the brain and the extensive neural death occurring first in the medial temporal lobe and the hippocampus, and later on in all of the gray matter.^2^ The underlying causes of AD remain unknown, and several hypotheses have been proposed for its origin. In favor of the hypothesis of amyloidosis is the fact that mutations in amyloid precursor protein (APP), or in the enzymatic components presenilin1/2 of gamma-secretase that cleaves out Aβ from APP, lead to higher Aβ levels and early onset of familial AD. The familial form of the disease is accompanied by metabolic dysfunction, inflammation and other symptoms.^3^ Regardless of its origin, the loss of memory function in AD is associated with synapse loss, even before any other pathology develops.^4,5^ Recent studies have shown impairments in neuronal network activity at the early stages of AD^6,7^ suggesting that synaptic dysfunction might be the earliest manifestation of AD.

Recent research suggests an Aβ-dependent neuronal hyperactivity and downregulation of glutamate receptors in mouse models of amyloidosis.^8^ Soluble Aβ species are believed to initiate pathological hyperactivity by suppressing glutamate reuptake, leading to excessive synaptic glutamate and hyperactivation of pre-existing active neurons.^9,10^ Furthermore, deficient glutamate transporter function has been observed in AD.^11^ This impairs the clearance of glutamate from the synaptic cleft, further contributing to excitotoxicity and neuronal damage. This interplay between Aβ, glutamate dysregulation, and neuronal hyperactivity has been reported for many years, however, focus on astrocytic synaptic function in early AD remains relatively unexplored.

Astrocytes have a crucial role in regulating the efficiency of synaptic transmission in the brain, where their activity directly impacts the cognitive performance and behavior of animal models.^12^ An important role of astrocytes in synaptic function comes from the uptake of glutamate from the synaptic cleft for conversion to l-glutamine, which is then shuttled back to the presynaptic neuron to generate new glutamate for neurotransmission.^13,14^ Previous reports have shown that synaptic coverage by astrocytic processes gives a cognitive advantage in hypomyelinated *shiverer* mice,^12^ and a direct correlation exists between the cognitive abilities of mammals and the size and complexity of its astrocytes.^15^ Astrocytes take up glutamate through glutamate transporters GLAST (glutamate/aspartate transporter, or excitatory amino acid transporter 1, EAAT1) and GLT-1 (glutamate transporter 1, or excitatory amino acid transporter 2, EAAT2).^16^ GLT-1 is the most abundant glutamate transporter in the brain, and it is expressed in astrocytes and synaptic terminals of neurons, however, the majority of GLT-1 protein in the whole brain seems located in astrocytes.^17,18^ GLT-1 is responsible for most of the glutamate clearance in brains of adult animals and it is crucial for synaptic neurotransmission and maintenance of glutamate levels below toxicity.^19–23^ GLT-1 deletion leads to spontaneous seizures^20^ and glutamate transporter dysfunction has an important role in glutamate excitotoxicity.^24^ An imbalance between the levels of astrocytic glutamate transporter GLT-1 related to glial fibrillary acidic protein (GFAP) expression has been reported in the brains of AD patients,^25^ however, the relationship between AD and astrocyte dysfunction in the brain is not yet fully understood.

Reactive astrogliosis in AD has been studied in the context of the presence of Aβ plaques. Several histopathological studies have found an accumulation of activated astrocytes surrounding Aβ plaques,^26^ and it has been suggested this is the origin of the inflammatory response in AD brains.^27^ Nevertheless, other studies suggest inflammation takes place before Aβ plaques have begun to aggregate in the brain.^28,29^ In the context of the tripartite synapse, astrocytes perform important synaptic functions, and astrocytic activation is likely to decrease their efficiency to support synaptic transmission by reducing the generation of important metabolites,^30^ and possibly through dysregulation of glutamate uptake.^31,32^ Nevertheless, the correlation between inflammation and loss of astrocyte-synaptic functions has been characterized mostly in vitro^33,34^ or in advanced stages of AD^35,36^ and early events of astrocyte-related synaptic dysfunction are less well-known.

To study the astrocytic synaptic function in the context of early AD, we took advantage of an *App* knock-in mouse model *App* ^*NL‑G‑F*^ which harbors the familial Swedish, Beyreuther/Iberian and Arctic mutations in the mouse *App* gene. This model avoids artifacts associated with APP overexpression and thus better replicates the human AD pathophysiology compared to models that overexpress APP. The *App* knock-in mice exhibit robust Aβ pathology, neuroinflammation, synaptic loss, and memory impairment.^37^ Here, using whole-cell patch-clamp recordings of synaptically activated glutamate transporter currents (STC) in astrocytes, we report that glutamate transporter function in hippocampal *App* ^*NL‑G‑F*^ mouse astrocytes is impaired, as reflected by the slow rate of glutamate clearance following synaptic stimulation of primary astrocytes. Since no changes in GLT-1 levels in either crude synaptosome preparations or FACS-isolated astrocytes were found, our findings cannot suggest a change in protein levels is responsible for the loss of function in glutamate uptake in the *App* ^*NL‑G‑F*^ mouse astrocytes. More importantly, decreased glutamate function from astrocytes could underlie many of the synaptic aberrations and cognitive dysfunction in *App* ^*NL‑G‑F*^ mice, suggesting that therapies aiming to restore synaptic function in AD should pay significant attention to astrocyte glutamate uptake.

## Material and Methods

### Animals and Husbandry

All experiments were performed under the ethical permit ID 407 granted by the Linköping animal research ethical board. The generation of *App* ^*NL‑G‑F*^ mice has been described before.^37^ The mice were bred and maintained in a 12–12 light-dark cycle and with ad libitum access to water and food, at Karolinska Institutet (Stockholm, Sweden). C57BL/6J mice were obtained from Janvier (Germany) and housed at Karolinska Institutet (Stockholm, Sweden). For this study, 6-month-old female mice were used for molecular biology, protein isolation, synaptosomal isolation and immunohistochemistry. For electrophysiology, we used 20 animals that started at 6 months of age but were processed one per day and reached 7.5 months old for the last experiments. Animals had access to food and water ad libitum and were housed in a 12-h light/dark cycle. Additional 6-month-old animals were used for, crude synaptosome preparation (5 *App* ^*NL‑G‑F*^ mice and 5 WT mice), and Aβ staining (2 *App* ^*NL‑G‑F*^ mice).

### Slice Preparation

For the preparation of acute slices, mice were deeply anesthetized with isoflurane and decapitated soon after the disappearance of corneal reflexes. For patch-clamp recordings, 300 μM thick horizontal slices were prepared from the hippocampus using a Leica VT1200 vibrating microtome (Leica Microsystems, Nussloch, Germany) in a dissection solution containing 250 mM sucrose, 2.5 mM KCl, 1.4 mM NaH_2_PO_4,_ 26 mM NaHCO_3,_ 10 mM glucose, 1 mM CaCl_2_ and 4 mM MgCl_2_ (310–330 mOsm) bubbled with carbogen gas (5% CO_2_, 95% O_2_). The recovery and recording aCSF solution contained 130 mM NaCl, 3.5 mM KCl, 1.25 mM NaH_2_PO_4_, 24 mM NaHCO_3_, 10 mM glucose, 2 mM CaCl_2_ and 1.3 mM MgCl_2_. After a minimum recovery period of 2 h, slices were transferred to a submerged recording chamber held at 32 ± 1 °C, with a perfusion rate of 2–3 mL per min with standard aCSF. Stimulation of brain slices with kainic acid (KA) was induced by continuous perfusion of 100 nM KA in aCSF in a recording chamber. The slices were allowed to stabilize for at least 30 min as previously described.^38^ These slices were also used for immunoblotting (WB) and immunohistochemistry (IHC).

### Immunohistochemistry

300-μm hippocampal coronal sections were postfixed overnight in 4% PFA and then stored at 4 °C. After washing with PBS (2 × 10 min) antigen retrieval was performed in 10 mM Tris, 1 mM EDTA, 0.05% Tween-20, pH 9.0 at 80 °C for 20 min. The slices were then washed for 5 × 10 min with PBS-T (PBS with 0.5% Triton X-100) and blocked using 10% normal goat serum (NGS) in PBS-T at room temperature for 5 h. Slices were incubated with primary antibodies, s100β (1:200, Abcam, ab4066, U.K.), GFAP (1:2000, Dako, Z0334, USA) and Aβ_x‑42_ (IBL, 18582, USA) for 48 h at 4 °C and Alexa Fluor-conjugated secondary antibodies (Invitrogen, Waltham, MA, USA) in the same blocking solution was performed overnight at 4 °C. After washing with PBS-T (5 × 10 min) and with PBS (1 × 10 min), slices were mounted in a mounting medium (Fluoromount-G, Thermo Fisher Scientific, 00-4958-02, USA). For Aβ_x‑42_ we conducted antigen retrieval with a formic acid treatment (using 90% formic acid for 5 min at 20–25 °C). For morphological analysis, GFAP and s100β, 25-μm thick z-stacks at 1-μm intervals were acquired in the stratum radiatum of the CA1 region in the hippocampus using LSM 880 confocal microscope (40× oil-objective, 16-bit, 1024 × 1024 pixels). All images were acquired with the same pinhole size that is equal to 1 airy unit (AU) for the longest wavelength with equal laser intensity, gain, and digital offset. 1–2 slices per animal were stained, and two to three images per slice were acquired without prior knowledge of Aβ aggregates and selected randomly. Morphology analysis was executed on Maximum Intensity Projections using Fiji software (National Institute of Health, Bethesda, MD, USA) with a Sholl analysis plug-in to assess GFAP-positive astrocyte branching. The center of the astrocytes was determined by s100β-positive soma. The number of the GFAP-positive processes at increasing radii from the soma (5–20 μm from soma at an interval of 5 μm) was automatically determined by the software. Soma size was measured by selecting the s100β-positive area by segmentation utilizing measurement tools implemented in the software previous pixel to micrometer calibration. The thickness of the branches was assessed manually at a 10 μm distance from the soma. For this analysis, we did not consider cells with cut branches that were interrupted or incompletely acquired, which generated a smaller data set than for soma size, where branches were not considered for the quantification. Lastly, GFAP intensity was measured by using whole images as regions of interest (ROI) and assessed by the measurement tools implemented in the software.

### Whole-Cell Patch-Clamp Recordings from Astrocytes

For patching astrocytes, borosilicate glass pipettes with a tip resistance of 5–7 MOhms were used. The glass pipettes were filled with a solution containing: 110 mM K-gluconate, 10 mM KCl, 4 mM Mg-ATP, 10 mM Na_2_-phosphocreatine, 0.3 mM Na-GTP, 10 mM 4-(2-hydroxyethyl) piperazine-1-ethanesulfonic acid (HEPES) and 0.2 mM ethylene glycol tetraacetic acid (EGTA) (pH 7.2–7.4; 270–290 mOsm). Astrocytes were visually identified by their small spherical shape and localization in the stratum radiatum and identity was confirmed by a hyperpolarized resting membrane potential, low input resistance and no voltage-dependent currents (see Figure 4a–c). Synaptic responses were evoked by electrical stimulation of the Schaffer collaterals (SC) using a bipolar concentric electrode (FHC Inc., Bowdoin, USA). The amount of current injected by the bipolar electrode was kept constant throughout the recordings from the same astrocyte. All recordings were made in the presence of picrotoxin (50 μM) to exclude the contribution of GABA_A_ receptors. The astrocytic current response to SC stimulation by a single constant current pulse was recorded in voltage-clamp at −80 mV. Astrocytic current to SC stimulation was recorded in the presence of glutamate receptor blockers NBQX and DL-AP5 and then in the presence of astrocyte glutamate transporter blocker TFB-TBOA (1 μM) together with the glutamate receptor blockers (NBQX and DL-AP5). Synaptically activated transporter-mediated currents (STCs) in astrocytes could then be isolated by subtracting the current recorded in the presence of TFB-TBOA (1 μM), from the total astrocytic current recorded earlier after SC stimulation in the same astrocyte (Figure 4g,j). To compare the astrocytic glutamate transporter function in the two strains, high-frequency stimulation (50 Hz) in the presence of NBQX and DL-AP5 was used and two trains were given of 9 pulses followed by 10 pulses. The astrocytic current response to stimulation by 9 and 10 pulses was then superimposed (Figure 4a,e) and the astrocytic current response to the 10th pulse was isolated.^39^ To isolate the STC, the astrocytic current in response to a single pulse stimulation in the presence of TFB-TBOA was subtracted from this 10th pulse (Figure 4b,f). All drugs were delivered through the perfusion of aCSF solution.

### Analysis of Patch-Clamp Recordings

All data were analyzed using the Clampfit 10.7 software (Molecular Devices, LLC, San Jose, CA, USA). The STC was best fitted to a single exponential function in clampfit^40^ and the decay time constant and the peak amplitude of the STC was obtained from this function. Astrocytic current responses to negative voltage steps of 1 mV were monitored throughout the experiment to ensure the quality of the recording and data analysis was done only for stable values (<20% variation). An average of 5 sweeps was used for all recordings.

### Extraction of Crude Synaptosomal Fractions

Hippocampi from *App* ^*NL‑G‑F*^ and WT mice (6 months old) were dissected from mouse brain and mechanically homogenized at 800 rpm with a glass-Teflon homogenizer in cold lysis buffer (1.5 M sucrose, 100 mM Hepes, and 10 mL Milli-Q H20 supplemented with phosphatase inhibitor cocktail and protease inhibitor 1:100, pH 7.5). The ratio of lysis buffer was 1 mL per 100 mg of tissue. Homogenates were centrifugated at 1000×g for 10 min at 4 °C and the supernatant was transferred to a new 2-ml reaction tube and the pellet was discarded (P1). The resulting supernatant was centrifugated at 12000×g at 4 °C for 20 min. The supernatant (S2) was immediately frozen, and the pellet (P2) which represents a crude synaptosomal preparation containing plasma membranes, and intracellular membranes including synaptosomes and mitochondria, was resuspended in 75 ul of RIPA buffer. P2 fractions were processed for Western blotting as described below.

### Immunoblotting

Western blot analysis was carried out as previously reported.^41^ Thick sections of the hippocampus were snap-frozen in liquid nitrogen. Solubilization of total protein was carried out using a detergent-based lysis buffer containing 40 mM Tris–HCl (pH 6.8), 1% NP-40, a protease inhibitor cocktail (Sigma-Aldrich) and phosphatase inhibitors (20 mM b-glycerophosphate, 2 nM okadaic acid, 50 mM NaF, 1 mM Na3VO4 from ROCHE) and the total amount of protein per sample was measured with a Pierce BCA kit (Thermo Fisher). An equal amount of protein (15 μg) was loaded on an SDS-PAGE gel and separated before transfer to a nitrocellulose membrane. Following the transfer skimmed milk-blocked blots were incubated with the following antibodies: mouse anti-GFAP (BD biosciences), and rabbit anti-Actin (Abcam), overnight. Secondary incubation was done using IR-Dye-coupled antirabbit or antimouse IgG at a 1:15000 dilution at room temperature in darkness (LI-COR). Immunoreactivity was detected by the Odyssey-IR detection system (LI-COR). ImageJ was used to perform densitometric analysis on our blots and normalized against GAPDH, actin, or α-tubulin signal to control protein loading in the membranes.

### Drugs

GABA_A_ receptor antagonist: PTX (Picrotoxin, 50 μM), AMPA receptor antagonist NBQX (1,2,3,4-Tetrahydro-6-nitro-2,3-dioxo-benzo­[f]­quinoxaline-7-sulfonamide hydrate; 10 μM), NMDA receptor antagonist DL-AP5 (DL-2-Amino-5-phosphonopentanoic acid; 50 μM), EAAT blocker TFB-TBOA ((3S)-3-3-4-(Trifluoromethyl) benzoyl amino phenyl methoxy-l-aspartic acid, 1 μM) and Kainic acid (KA), were obtained from Tocris Bioscience (Bristol, U.K.).

### Cell Isolation

Cell isolation from mouse brain tissue was performed as previously reported (Hasel et al., 2021). Briefly, 6 months-old *APP^NL‑G‑F^* and wild-type mice were sacrificed by decapitation and the brains were immediately put into PBS. Cortices were dissected from the rest of the brain, cut and diced into small pieces and transferred into enzyme solution containing 1× EBSS (#14155048, Thermo Fisher), 0.46% glucose (#G8270, Sigma-Aldrich), 26 mM NaHCO_3_(#S5761, Sigma-Aldrich), 0.5 mM EDTA (#E9884, Sigma-Aldrich), containing 200 U of papain (#LS003126, Worthington Biochemical) per brain. The tissue was then incubated at 37 °C for 40 min inverting the tube for mixing purposes every 10 min. After the enzymatic digestion, the brain tissue was transferred to a canonical tube and washed with a trypsin inhibitor solution three times 1× EBSS, 0.46% glucose, 26 mM NaHCO_3_ containing 1 × low-ovomucoid solution (10× stock: 15 mg/mL of BSA (#0332, VWR) and 15 mg/mL of ovomucoid (#LS003086, Worthington Biochemical)). All the remaining steps were performed on wet ice. After the last wash, the tissue was triturated using a serological pipet. This step was repeated 3 times, letting the nonhomogenized tissue settle every time. The dissociated cells were then carefully transferred to a new tube containing high ovomucoid solution (1× EBSS, 0.46% glucose, 26 mM NaHCO_3_ containing 1 × high-ovomucoid solution (10× stock: 30 mg/mL of BSA and 30 mg/mL of ovomucoid). The cell suspension was centrifuged at 400*g* for 5 min at 4 °C and the pellet was resuspended in PBS (#14190169, Thermo Fisher). The cell suspension was then passed through a 20 μm cell strainer (#43-50020-03, pluriStrainer). The collected solution was centrifuged at 400*g* for 5 min at 4 °C and the pellet containing the cells was resuspended in 500 μL of 1% BSA in PBS.

### Fluorescence Activated Cell Sorting of astrocytes

Cells were sorted on a FACSAria III Cell Sorter (BD Biosciences). Six-month-old *APP^NL‑G‑F^* and wild-type mice were used for sorting, and gates were set to collect single cells that were DAPI (#D1306, Thermo Fisher Scientific) negative and GLAST (ACSA-1, APC, #130-123-555, Miltenyi Biotec) positive. About 1 million GLAST-positive cells were collected for each sample into PBS containing 1% BSA.

### Statistics

A specific statistical analysis was used for each type of experiment to fit the experimental design. Briefly, when only two groups were compared (immunostaining, Western blots, synaptosome densitometry, astrocyte input resistance and glutamate uptake quantification as a measure of time decay and measured glutamate amplitude), a nonparametric unpaired Mann–Whitney test was used to establish differences between WT and *APP^NL‑G‑F^* groups. For the Sholl analysis in Figure 1b, two-way ANOVA repeated measures followed by a posthoc multiple Bonferroni test were used to compare means with distance to the soma of the astrocyte. Each figure defines the biological *n* numbers in the legend. For most figures, each data point represents an individual animal, a different cell measured, or an independent cell culture experiment.

**Figure 1:**
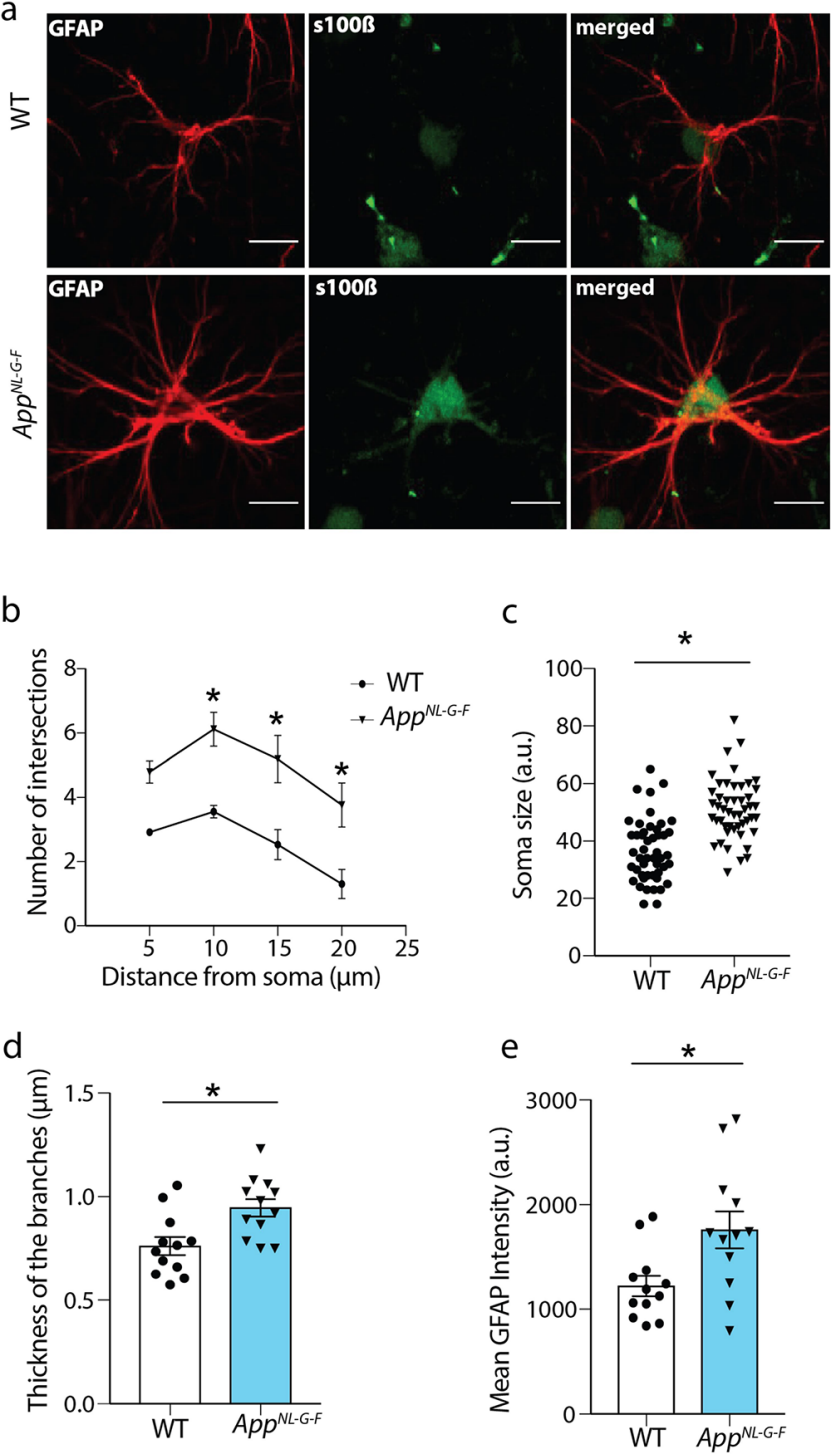
Astrocytes in the stratum radiatum of the CA1 region from *App* ^*NL‑G‑F*^mice have increased branching and soma size. (a) Immunostaining of hippocampal sections from *App* ^*NL‑G‑F*^ and WT mice with antibodies against GFAP (red) and s100β (green) to visualize processes and cell soma, respectively. Scale bar = 10 μm. (b) Scholl analysis, where the number of GFAP-positive processes is shown at 5, 10, 15, and 20 μm distance from the soma. Astrocytes in the *App* ^*NL‑G‑F*^ mice have a significantly increased number of branches compared to WT, shown as mean number ± SEM at specific distances (two-way ANOVA; *p* < 0.0001 for distance and *p* = 0.0175 for genotype; with Sidak′s multiple comparisons at 5, 10, 15, and 20 μm distances; *p < 0.05, adjusted *p* = 0.08, 0.01, 0.008, 0.01, respectively; *n* = 36 cells from 4 *App^NL‑G‑F^* and 3 WT mice). (c) Soma size in arbitrary units (au), measured as s100β-positive areas, is significantly bigger in *App^NL‑G‑F^* mice compared to WT group (two-tailed *t* test; *p < 0.0001, *n* = 4 *App^NL‑G‑F^*, 3 WT mice, based on average of at least 12 cells per animal). (d) Thickness of the branches, calculated manually at 10 μm distance away from soma (two-tailed *t* test; *p = 0.01, 2 cells per animal, and 3 branches per cell is measured for 4 *App^NL‑G‑F^* and 3 WT mice). (e) Mean GFAP intensity in arbitrary units, measured as defining the whole image as a region of interest (two-tailed *t* test; *p = 0.008, *n* = 12 images/group). Bar graph showing mean ± SEM.

## Results

### Astrocytes in *App* ^*NL‑G‑F*^ Mice Show an Increase in Thickness and Number of Processes, Soma Size and GFAP Intensity

Activated astrocytes have previously been reported in the *App* ^*NL‑G‑F*^ mice,^37^ however, a detailed characterization of astrocytes in this mouse model is still lacking. Hence, we performed a morphological study of astrocytes analyzing the number and thickness of processes, soma size and GFAP intensity in the *App* ^*NL‑G‑F*^ mice compared to WT controls. Astrocytes were stained in the stratum radiatum of the hippocampus with antibodies against GFAP and cytosolic calcium-binding protein s100β (Figure 1a). Astrocytes in the *App* ^*NL‑G‑F*^ mice showed an increase in GFAP-positive branches (Figure 1b; Two-way ANOVA, *p* < 0.05), larger soma size (Figure 1c; 50.94 ± 1.51 μm in *App* ^*NL‑G‑F*^ vs 36.6 ± 1.56 μm in WT mice, two-tailed unpaired *t* test, *p* < 0.05) and greater thickness (Figure 1d; 0.946 ± 0.042 μm in *App* ^*NL‑G‑F*^ vs 0.762 ± 0.04 μm in WT, Two-tailed Unpaired *t* test, *p* < 0.01). Also, the mean GFAP intensity was increased in *App* ^*NL‑G‑F*^ mice compared to the WT group (Figure 1e; 1760 ± 175.4 in *App* ^*NL‑G‑F*^ vs 1222 ± 97.27,a.u.); Two-tailed Unpaired *t* test, *p* < 0.05). Thus, astrocytes in the hippocampus of *App* ^*NL‑G‑F*^ mice display a reactive morphology. Animals in this study have small Aβ aggregates dispersed through the hippocampus (Figure S7a), however at this stage, we could not see plaque-surrounding astrocytosis (Figure S7b).

### GFAP but not GLT-1 are Elevated in the Hippocampus of *App* ^*NL‑G‑F*^ Mice

To further confirm the increase in GFAP levels in the *App* ^*NL‑G‑F*^ mice compared to WT mice, we used Western blot analysis of hippocampal homogenates, normalized to GAPDH levels. We found GFAP to be increased in the *App* ^*NL‑G‑F*^ mice (*App* ^*NL‑G‑F*^ *=* 174.6 ± 14.2 vs WT = 100 ± 7.3, *n* = 5 per group, **p = 0.007, Figure 2a,c). The glutamate transporter protein GLT-1 was however unchanged in *App* ^*NL‑G‑F*^ mice compared to WT counterparts (WT = 100 ± 21.6 vs *App* ^*NL‑G‑F*^ = 159.9 ± 24.16, *n* = 5 per group, *p* = 0.952, Figure 2a,b). When we artificially increase extracellular glutamate by adding 100 nM kainic acid to slices from WT mice, the levels of GLT-1 were significantly increased (WT = 100 ± 7.27, *n* = 3; WT KA+=172.1 ± 15.07, *n* = 3; *p* = 0.05). This increase was not seen in the, *App* ^*NL‑G‑F*^ where the baseline level of GLT-1 is already at the same level as in the KA-treated WT slices (Figure S2), while no changes were observed for GLAST after KA stimulation for WT samples (WTKA- = 100 ± 3.28, *n* = 6; WTKA+ = 108.5 ± 3.83, *n* = 6; *p* = 0.12) nor for *App* ^*NL‑G‑F*^ samples (*App* ^*NL‑G‑F*^ KA- = 100 ± 4.9, *n* = 7; *App* ^*NL‑G‑F*^ KA- = 95.54 ± 9.87, *n* = 7; *p* = 0.69).

**Figure 2:**
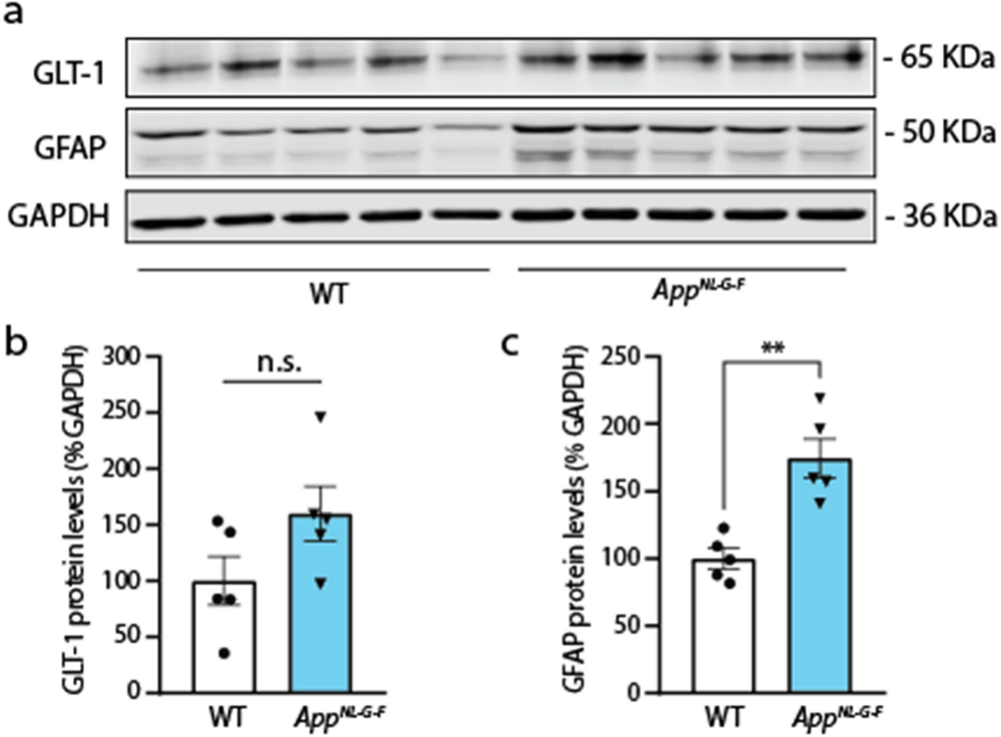
*App* ^*NL‑G‑F*^ shows increased GFAP levels but normal GLT-1 levels in whole hippocampus lysates. (a) Western blot analysis of whole hippocampus lysates from *App* ^*NL‑G‑F*^ and WT mice. (b) Densitometric analysis of synaptosome enriched fractions shows that GLT-1 protein levels do not change significantly in *App* ^*NL‑G‑F*^ compared to WT mice (*n* = 5 animals per group; *n.s*. = 0.09). (c) Densitometric analysis of GFAP protein levels in FACS-isolated hippocampal astrocytes from *App* ^*NL‑G‑F*^ and WT mice (*n* = 5 animals per group; *p = 0.007).

### Synaptosomal GLT-1 is Unchanged in *App* ^*NL‑G‑F*^ Mice

To test whether the increased levels of GLT-1 in astrocytes as seen in brain slices and cultures are also reflected in membrane-bound GLT-1, we prepared crude synaptosome-enriched fractions from *App* ^*NL‑G‑F*^ mice (Figure 3). Synaptosome-enriched (P2) fractions showed higher amounts of synaptic proteins such as PSD95 and synaptophysin, while also displaying enrichment of GLT-1 compared to the S2 fraction containing mostly cytosolic proteins (Figure 3a). When compared, synaptosome-enriched fractions showed no significant change in the levels of GLT-1 protein between *App* ^*NL‑G‑F*^ and WT mice independently of the housekeeping gene used for normalization (Tubulin normalization, WT = 100 ± 10.7; *App* ^*NL‑G‑F*^ = 146.8 ± 41.55; *p* = 0.84; Figure 3b,c). Levels of GFAP protein between *App* ^*NL‑G‑F*^ and WT mice were not significantly changed (Tubulin normalization, WT = 100 ± 6.52; *App* ^*NL‑G‑F*^ = 198.1 ± 33.79; *p* = 0.055; Figure 3d), neither the ratio between GLT-1 and GFAP relative protein levels comparing WT and *App* ^*NL‑G‑F*^ mice (WT = 120.9 ± 16.34; *App* ^*NL‑G‑F*^ = 118.2 ± 59.17; *p* = 0.22; Figure 3e).

**Figure 3:**
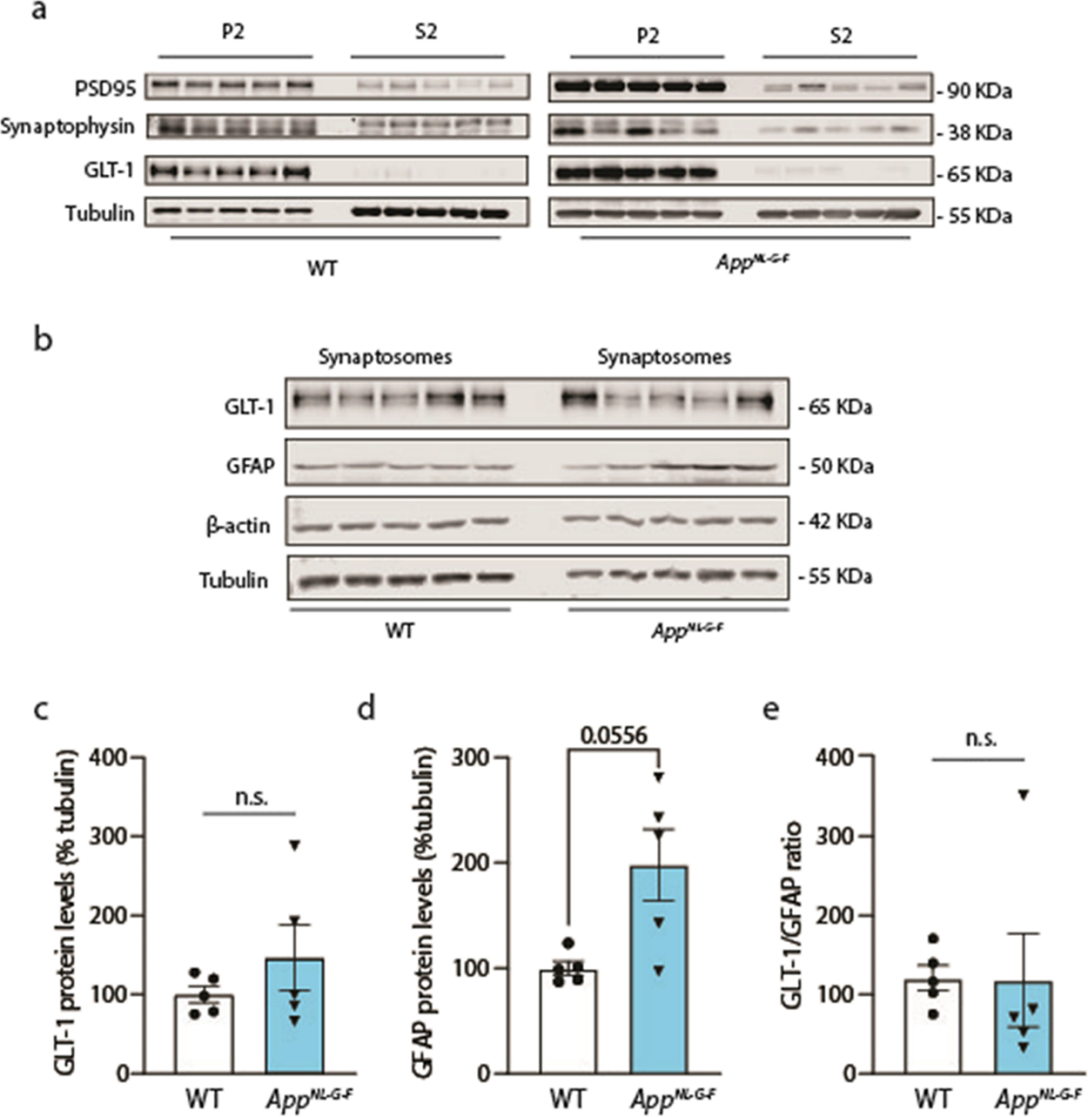
Astrocytic GLT-1 is unchanged in crude synaptosomal preparation of *App* ^*NL‑G‑F*^ mice. (a) Western blot analysis of these synaptosome enriched fractions from *App* ^*NL‑G‑F*^ and WT mice. P2 corresponds to the crude synatptosomal preparation, while S2 is the supernatant fraction containing cytosolic proteins. (b) Western blots of synaptosomal enriched fractions showing protein expression of GLT-1 and PSD95 and housekeeping genes β-actin and tubulin. (c) Densitometric analysis of synaptosome enriched fractions shows that GLT-1 protein levels are not significantly changed in *App* ^*NL‑G‑F*^ compared to WT mice (*n* = 5 animals per group; *n.s.* = 0.69). (d) Densitometric analysis of GFAP protein levels in synaptosome enriched fractions shows no significant difference between *App* ^*NL‑G‑F*^ and WT mice (*n* = 5 animals per group; *n.s*. = 0.055). (e) Ratio of relative abundance of GLT1 over GFAP protein levels between *App* ^*NL‑G‑F*^ and WT mice (*n* = 5 animals per group; *n.s*. = 0.22).

### GLT-1 is Unchanged in FACS-Isolated *App* ^*NL‑G‑F*^ Isolated Astrocytes

To measure GLT-1 levels directly in hippocampal astrocytes, we used FACS to separate astrocytes expressing the APC antigen. After sorting, we tested approximately one million astrocytes per animal using Western blot analysis to measure GLT-1 levels and found no differences between *App* ^*NL‑G‑F*^ and WT-isolated astrocytes (Tubulin normalization, WT = 100 ± 29.7; *App* ^*NL‑G‑F*^ = 74 ± 10.95; *p* = 0.44; Figure S2a,b). Concurrent with morphological changes, GFAP was significantly increased in *App* ^*NL‑G‑F*^ astrocytes compared to WT animals (Tubulin normalization, WT = 100 ± 7.7; *App* ^*NL‑G‑F*^ = 131 ± 8.7; *p* = 0.03; Figure S2a,c).

### Astrocytic Passive Membrane Properties are Changed in *App* ^*NL‑G‑F*^ Mice

To understand if the astrocytic reactivity shown by morphology and protein levels is reflected in the astrocytic membrane properties; membrane potential, input resistance and the voltage–current response of astrocytes were recorded in acute brain slices from *App* ^*NL‑G‑F*^ mice and compared to wild-type controls. Both groups displayed the typical hyperpolarized RMP (−76.44 mV ± 0.90, *n* = 9 in WT and −73.83 mV ± 1.55, *n* = 9 in *App* ^*NL‑G‑F*^; Figure 4a). Interestingly, the mean RMP, though not statistically significant (*p* = 0.16, unpaired *t* test), was slightly depolarized in *App* ^*NL‑G‑F*^ mice compared to WT. The input resistance of astrocytes was significantly higher in *App* ^*NL‑G‑F*^ mice (13.24MOhms ± 0.86, *n* = 9) compared to WT (10.72MOhms ± 0.54, *n* = 9, *p* < 0.05, unpaired *t* test; Figure 4b) and the IV plot showed a shift in the astrocytic membrane potential response to increasing current injections in *App* ^*NL‑G‑F*^ (*n* = 9) versus WT (*n* = 9; Figure 4c) suggesting that the passive membrane properties of astrocytes in *App* ^*NL‑G‑F*^ mice are altered compared to WT. One of the most important functions of astrocytes is the uptake of glutamate after synaptic transmission by astrocytic glutamate transporters. The glutamate uptake by astrocytes is electrogenic and hence can be recorded as synaptically activated transporter-mediated current (STC). To isolate the STC, the astrocytic current response to a single pulse stimulation of SC was recorded in the presence of AMPA and NMDA receptor blockers NBQX and AP5 and then in the presence of astrocytic glutamate transporter blocker TFB-TBOA. Isolation of STC was performed by subtracting the TFB-TBOA sensitive current from the total inward current. The decay kinetics of the STC shows the time course of glutamate clearance by the astrocyte.^40^ The STC decay was best fitted to a single exponential function and the decay time constant and the peak amplitude were calculated. The STC decay time constant though not statistically significant showed an increase from 6.483 ms ± 0.9304 (*n* = 5; Figure 4k) in WT compared to 8.479 ms ± 1.232 in *App* ^*NL‑G‑F*^ mice (*n* = 4, Figure 4k). Interestingly, a single pulse stimulation in *App* ^*NL‑G‑F*^ mouse was not able to induce detectable STC in some of our recordings and therefore the corresponding value in *App* ^*NL‑G‑F*^ could not be calculated for those. However, STC was detected from the same astrocytes at higher stimulations suggesting the impairment in their glutamate uptake machinery. We found the peak amplitude in *App* ^*NL‑G‑F*^ mice −5.40 ± 2.77 (*n* = 4) significantly decreased compared to −12.21pA ± 1.15 in WT mice (*n* = 5; Figure 4l) suggesting impaired glutamate uptake.

**Figure 4:**
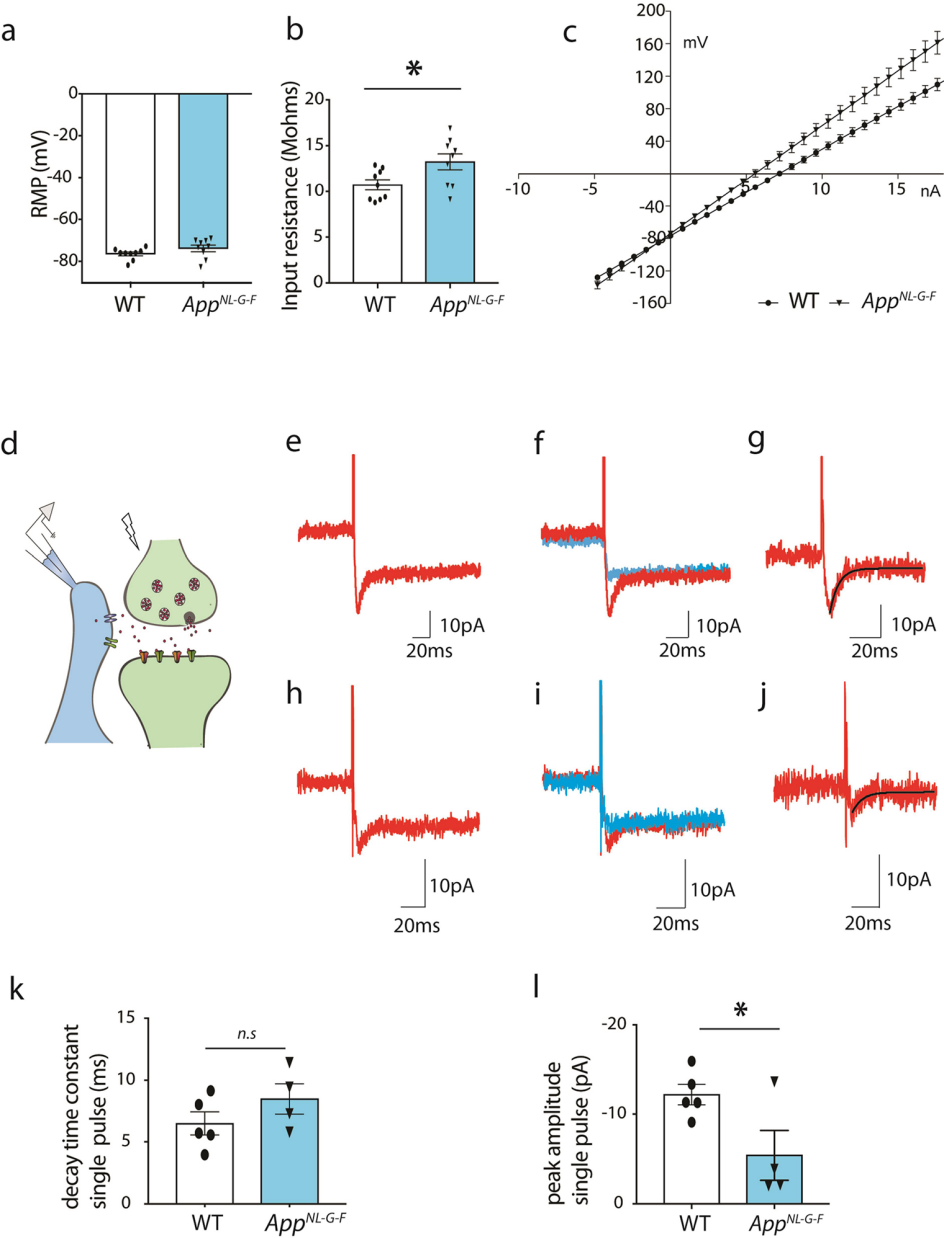
Passive membrane properties of astrocytes are altered in *App* ^*NL‑G‑F*^mice compared to WT mice. (a) Resting membrane potential (RMP) of astrocytes in *App* ^*NL‑G‑F*^ mice (*n* = 9) is not significantly different compared to WT (*n* = 9, *p* = 0.16, unpaired *t* test). (b) *App* ^*NL‑G‑F*^ (*n* = 9) astrocytes have significantly higher input resistance than WT (*n* = 9, *p = 0.02, unpaired *t* test), (c) *I*–*V* plot showing a shift in astrocyte voltage response to current injection in WT hippocampal slices (*n* = 9, red) compared to *App* ^*NL‑G‑F*^ (*n* = 9, green). (d) Scheme illustrating the setup for recording synaptically activated glutamate transporter current (STC). Schaffer collaterals (SC) were stimulated using a bipolar electrode leading to glutamate release. Representative trace showing astrocyte current response (red) to single pulse SC stimulation in the presence of glutamate receptor blocker (NBQX, AP5) in (e) WT and (h) *App* ^*NL‑G‑F*^ mice. Representative trace showing astrocyte current response to single-pulse SC stimulation (red) superimposed over the astrocytic current response in the presence of glutamate transporter blocker (TFB-TBOA, blue) in the same astrocyte in (f) WT and (i) *App* ^*NL‑G‑F*^ mice. STC peak amplitude and decay time constant were obtained after best fitting to a single exponential function (black line) in (g) WT and in (j) *App* ^*NL‑G‑F*^ mice. (k) STC decay time constant in WT (*n* = 5) and *App* ^*NL‑G‑F*^ mice (*n* = 4, *p* = 0.22, n.s.= non significant, unpaired *t* test) (l) STC peak amplitude significantly decreased in *App* ^*NL‑G‑F*^ mice (*n* = 4) compared to WT (*n* = 5, *p = 0.003, unpaired *t* test). Bars show mean ± SEM.

### Glutamate Uptake by Astrocytes is Impaired in *App* ^*NL‑G‑F*^ Mice

The initial recordings with a one-stimulation pulse suggested impairment in glutamate uptake in astrocytes in the *App* ^*NL‑G‑F*^ mice. To further assess astrocyte glutamate transporter function we increased the stimulation to a train of 9 pulses followed by a train of 10 pulses of 50 Hz. The astrocyte current response was recorded under both these conditions in the presence of AMPA and NMDA receptor blockers NBQX and AP5 respectively. The astrocytic current response to the 10th pulse was obtained by subtracting the response of 10 pulses from that of 9 pulses. We then recorded the current response to specific astrocytic glutamate transporter blocker TFB-TBOA from the same astrocyte. This TFB-TBOA insensitive current was subtracted from the astrocytic current response to the 10th pulse to isolate the STC (Figure 5d,h). The STC decay was fitted to a single exponential function and the decay time constant and peak amplitude were calculated. The decay time constant was significantly increased in *App* ^*NL‑G‑F*^ mice (10.74 ms ± 1.348, *n* = 6) compared to that of WT mice (6.717 ms ± 0.673, *n* = 5, *p* < 0.05, unpaired *t* test; Figure 5i). The peak amplitude of the STC in *App* ^*NL‑G‑F*^ mice (−9.96 pA ± 3.84, *n* = 6) was also found to be significantly decreased compared to that of WT mice (−31.61 pA ± 3.71, *n* = 5, *p* < 0.01, unpaired *t* test; Figure 5j). Thus, although *App* ^*NL‑G‑F*^ mice exhibit an increase in the expression levels of GLT-1 (Figure 5b,d), the rate of glutamate clearance is as indicated by the STC decay constant and STC peak amplitude is severely impaired in *App* ^*NL‑G‑F*^ mice. Our results show that astrocytic glutamate transport and their membrane proteins are severely affected in this mouse model of AD.

**Figure 5:**
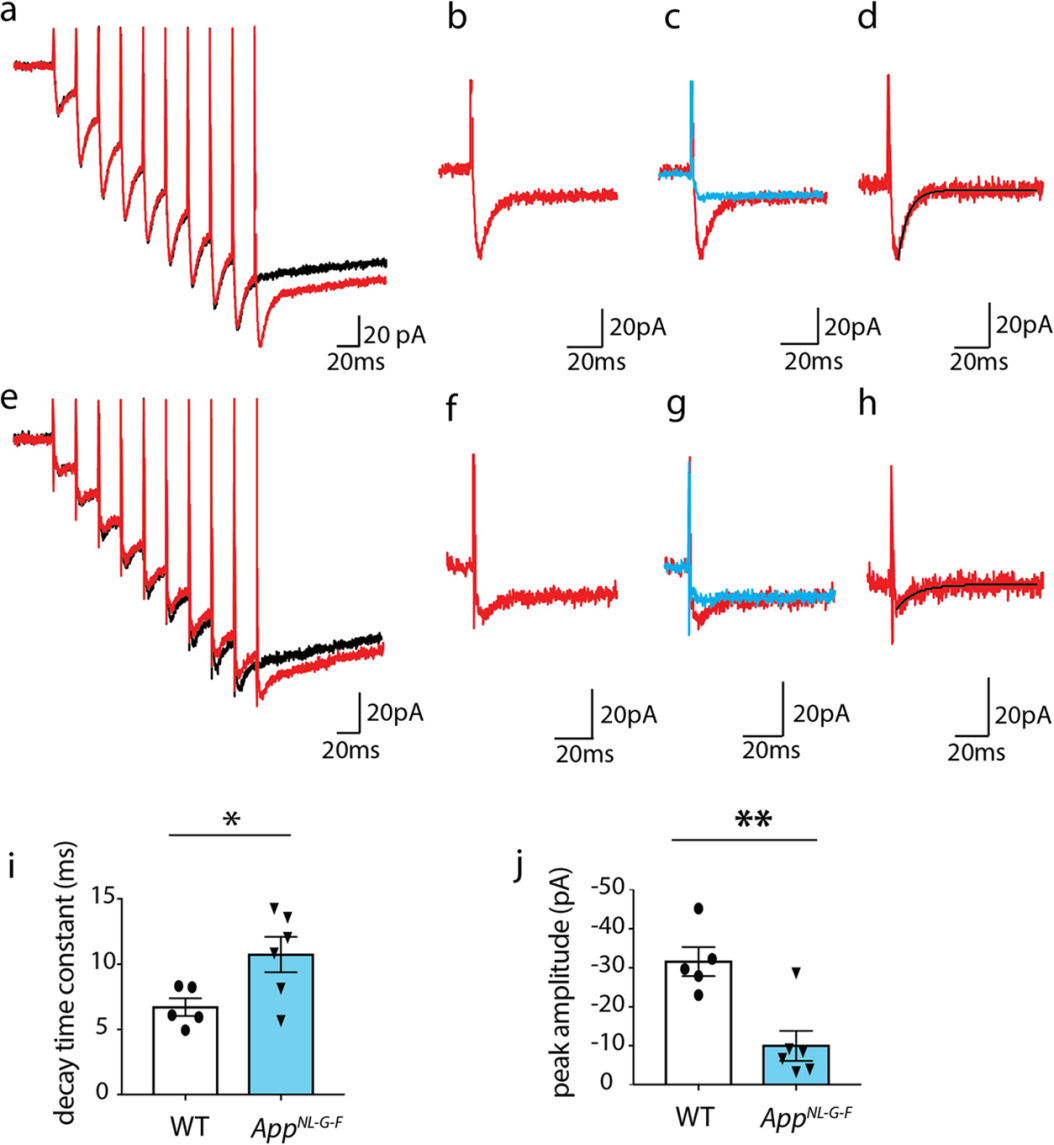
Astrocytes in *App* ^*NL‑G‑F*^ mice exhibit a reduced rate of glutamate clearance compared to WT mice. Representative superimposed traces showing the astrocytic current recording after 9 (black) and 10 pulses (red) of 50 Hz SC stimulation in the presence of NBQX and AP5 in (a) WT and (e) *App* ^*NL‑G‑F*^ mice. The resultant trace showing the astrocytic response to the 10th pulse was obtained by subtracting the response of 9 pulses from 10 pulses in (b) WT and (f) *App* ^*NL‑G‑F*^ mice. Representative traces showing the response of TFB-TBOA (blue) in the same astrocyte superimposed over the astrocytic current obtained due to the 10th pulse in (c) WT and (g) *App* ^*NL‑G‑F*^ mice. STC was isolated by subtracting the response of TFB-TBOA from the 10th pulse. This isolated STC was best fitted by a single exponential function (black line) to obtain the decay time constant (d) WT (h) *App* ^*NL‑G‑F*^ mice and peak amplitude of STC. (i) Decay time constant of the STC by the 10th pulse, which indicates the rate of glutamate clearance, was significantly increased in *App* ^*NL*‑*G‑F*^ mice (*n* = 6) compared to WT (*n* = 5, unpaired *t* test, *p = 0.0339) whereas (j) peak amplitude of STC by was significantly lower in *App* ^*NL‑G‑F*^ mice (*n* = 6) vs WT (*n* = 5, unpaired *t* test, *p = 0.003). Bars show mean ± SEM.

The reduction of the glutamate transporter current in the *App* ^*NL*‑*G‑F*^ mice as compared to WT, suggests that the transporter does not form a part of the functional synapse, evidenced by the data from synaptosomes and the glutamate transporter current experiments. Also, alterations in GLT-1 regulation could be involved in the lack of glutamate uptake by *App* ^*NL*‑*G‑F*^ astrocytes. This hypothesis is supported by our results showing that, when we artificially increase extracellular glutamate by adding 100 nM kainic acid to slices from WT mice, the levels of GLT-1 were significantly increased (WT = 100 ± 7.27, *n* = 3; WT KA+ = 172.1 ± 15.07, *n* = 3; *p* = 0.05).

## Discussion

Astrocytes perform several important functions in the brain such as maintaining ionic homeostasis, protecting the CNS against insults in case of injury or inflammation and also participating in the energy metabolism of the neurons and synaptic transmission.^42^ These functions are compromised in neurodegenerative diseases such as AD, where astrocytes show reactivity, with changes in expression levels of various proteins and functional impairment.^43^ Recent studies have suggested a causative relationship between reactive astrocytes and impaired memory in animal models of AD.^27^ However, most AD animal models use overexpression of amyloid precursor protein (APP) which leads to overexpression of other APP fragments that can have uncharacterized signaling activity.^44^ Experimental data based on these models can be misleading and difficult to translate to humans. In this study, we used the novel *App* ^*NL‑G‑F*^ knock-in mouse model which expresses Aβ42 with the Arctic mutation along with the Swedish and Beyreuther/Iberian mutations and exhibits Aβ pathology, neuroinflammation and memory impairments.^37^

We found that *App* ^*NL‑G‑F*^ astrocytes show reactive features with increased GFAP intensity, branch thickness and swollen cell bodies (Figure 1). As previously reported, we also found elevated protein levels of GFAP in the hippocampus of *App* ^*NL‑G‑F*^ mice (Figure 2), suggesting astrocytic activation. This is especially important in our context because it supports other reports showing a relationship between astrocyte activation and loss of synaptic function^45,46^ *·* ^25,39,47,48^ We show here that astrocytic membrane passive properties are affected in the *App* ^*NL‑G‑F*^ astrocytes with an increased input resistance and a shifted current/voltage response profile (Figure 4). These changes in astrocyte membrane properties likely affect their function including glutamate uptake, but also gliotransmitter release, as similar mechanisms were described for hippocampal astrocytes in rats.^49^

Glutamate transport through the astrocytes is necessary for the proper functioning of synaptic transmission and to prevent postsynaptic overexcitation.^50,51^ In adult animals, most of the brain GLT-1 and GLAST glutamate transporters are located in astrocytes,^19,52–54^ and the same holds for the hippocampus.^55^ A quantitative study of glutamate uptake in hippocampal synaptic terminals has shown that GLT-1 protein density is 10 times higher in astroglial membranes than in neuronal spines^56^ and several single-cell sequencing analyses show that GLT-1 is prominently expressed by GFAP positive astrocytes.^57,58^ The expression of glutamate transporters is altered in AD and Aβ has been shown to affect the expression pattern of glutamate transporters,^59^ making them important candidates for understanding the mechanisms underlying AD. Nevertheless, when we looked at GLT-1 expression it was not changed in hippocampal lysates (Figure 2). When exposed to glutamatergic stimulation, WT hippocampal slices do regulate GLT-1 levels but not GLAST levels and the GLT-1-specific regulation is lost in *App* ^*NL‑G‑F*^ mice (Figure S2), suggesting that GLT-1 regulation could be affected by increased Aβ levels. To understand if this change in levels had functional relevance in the hippocampus, we recorded glutamate transporter currents in this *App* ^*NL‑G‑F*^ model.^60^ Using electrophysiology, we observed a significant impairment in glutamate transporter function. The impairment was so severe that the glutamate transporter current was not detectable with a single stimulation in the *App* ^*NL‑G‑F*^ mice (Figure 4) and with repetitive stimulation, the decay time constant increased because of accumulated extrasynaptic glutamate (Figure 5).

When we prepared crude synaptosomal fractions and tested FACS-isolated astrocytes for GLT-1 protein levels, we saw no differences between *App* ^*NL‑G‑F*^ and WT mice. The evidence from our crude synaptosomal preparations of *App* ^*NL‑G‑F*^ mice in correlation with our electrophysiological recordings suggests that the increase in GFAP expression and lack of GLT-1 recapture function might be correlated (Figure 3). This effect could be further exacerbated by the swelling of the astrocytes which involves a retraction from the synaptic cleft and thus reduced access to it.^61,62^ FACS-isolated astrocytes from *App* ^*NL‑G‑F*^ mice do show increased GFAP expression (Figure S1), consistent with astrocyte reactivity to elevated Aβ in the brain. Nevertheless, there is a lack of correlation of GLT-1 levels relative to GFAP also seen in our synaptosome results, which suggests the glutamate uptake dysfunction in the mutant mice could be more related to delocalization at the synapse rather than GLT-1 regulation. Several studies have reported a reduction of GLT-1 in the brain of AD patients,^11,36,63^ however all of these reports used post-mortem AD brains with the majority of samples at Braak stages V and VI, thus downregulation of GLT-1 observed here can be a consequence of extensive neurodegeneration in the brain. Mouse models with AD mutations have shown an inverse correlation between Aβ levels and GLT-1 function,^64,65^ however, all of these models had overexpression of familial AD mutations, thus, leading to severe plaque accumulation and neural dysfunction, which must be considered with caution when translating to clinical AD.

Other reports have tried to test a therapeutic approach for GLT-1, where they compensated for the loss of GLT-1 function by overexpression, which led to the restoration of cognitive function in APP_Sw,Ind_ mice;^66^ however, the therapeutic mechanism remained speculative and based on GLT-1 downregulation as the main driver of the pathological phenotype. In rats, the pharmacological blockade of GLT-1 generates memory impairment without decreasing GLT-1 levels,^67^ which is closer to the phenotype we observed in our experiments. It is important to consider the possibility that Aβ can directly affect the glutamate transporter function in several ways. Previous evidence shows that Aβ_1–42_ can delocalize GLT-1 in astrocytes, impairing clearance of synaptically released glutamate.^68^ These results are directly per our observations, where increased Aβ production in the *App* ^*NL‑G‑F*^ mice at 6 months of age induces the loss of astrocytic glutamate uptake in postsynaptic membranes. On the other hand, the proinflammatory effects of Aβ cannot be overlooked: our work and others^37,69^ have reported a prominent pro-inflammatory profile of astrocytes in *App* ^*NL‑G‑F*^ mice, with a report showing astroglial gene expression in our model that is very close to expression profiles found in human AD brains.^70^ Also, we observe dysregulation of GLT-1 expression in our astrocytic primary cultures, where GLT-1 appears upregulated in *App* ^*NL‑G‑F*^ astrocytes, however cultured astrocytes downregulate GLT-1 in the absence of neurons,^71,72^ thus we cannot rule out the possibility that Aβ is inhibiting this downregulation in *App* ^*NL‑G‑F*^ astrocytes, making GLT-1 appear increased. Our general hypothesis is that Aβ alters the GLT-1 expression profile acutely (as shown in cultures) and chronically (as shown in 6 m.o. animals) in astrocytes through many uncharacterized mechanisms and regulators. It is possible that the swelling of the astrocytic cell body and processes (as seen in Figure 1) induces a retraction of fine astrocytic processes from the synapses. The astrocytes thus are not positioned to take up glutamate released from the synapses.^62,73,74^ Lastly, our synaptosome results might be explained by impairment of the ubiquitination system that regulates GLT-1 turnover,^75^ or by defects in autophagy, which have been previously reported for this mouse model;^76^ yet, we have not seen GLT-1 accumulation in our cytoplasmic fractions during subcellular fractionation to suggest this could be the case.

The rate of glutamate clearance as calculated by the method used here depends on the electrotonic properties of the astrocytic membrane.^40^ The change in membrane properties in the *App* ^*NL‑G‑F*^ mice is not enough to solely explain the extensive reduction in glutamate transporter current. Hence, we propose that the membrane properties of the astrocytes and the glutamate transporter function are altered in *App* ^*NL‑G‑F*^ mice. Glutamate excitotoxicity is one of the many mechanisms contributing to neurodegeneration in AD,^77–80^ and it occurs when excess glutamate activates extrasynaptic glutamate receptors in the postsynaptic neuron. The diminished capability of astrocytes to recapture glutamate can contribute to this effect, as shown before for EAAT2b splicing variants with reduced glutamate transport rates.^36^ There are studies suggesting neurons have functional GLT-1 and actively participate in glutamate uptake;^17,43,63^ , although glutamine synthetase is expressed mainly in astrocytes, and its function in these glial cells is essential for life.^36^ Also, altered glutamatergic function with normal levels of GLT-1 has been reported before for APP/PS1 mice.^81^ One hypothesis suggested for this phenomenon is that Aβ could block GLT-1 activity in a way that resembles selective channel inhibitors,^9,82^ although the mechanism remains unknown.^83,84^

Most of the pharmacological treatments for AD have targeted neurons but the important role played by astrocytes in AD makes them interesting candidates for therapies,^85,86^ especially considering the importance of glutamate reuptake for brain function.^87,88^ However, increasing the expression of glutamate transporters alone might not be enough to restore the proper functioning of astrocytes as it does not guarantee functional localization at the synapses since the astrocyte remains reactive. Instead, the focus should be on restoring astrocytes’ health and synaptic expression profile. Membrane properties, expression levels of GFAP and related cascades, branching, and synaptic astrocytic activity should be considered when designing treatments for AD, as these factors are important for efficient astrocytic glutamate uptake and proper neurotransmission in the brain.

## Supplementary Material



## Data Availability

Data for this work is archived and publicly available upon request at the Karolinska Institutet repository and the Swedish National Archive. AI tools have not been used in the writing of this manuscript.

## Notes

Additional experimental details, materials, and methods, including NL-G-F FACS isolated astrocytes and brain slices Western blots as well as immunostaining of brain slices for β-amyloid and FACS records of all fractions collected (PDF)
