# Supplementary material for: Reactive Astrocytes with Reduced Function of Glutamate Transporters in the App NL‑G‑F Knock-in Mice
Source: ACS Chem Neurosci. 2025 May 27;16(11):2035–47. doi: 10.1021/acschemneuro.4c00714 (PMC12142577; doi:10.1021/acschemneuro.4c00714)
Supplement: Supplementary file 1 [file cn4c00714_si_001.pdf]

SUPPLEMENTARY INFORMATION

Title: **Reactive astrocytes with reduced function of glutamate transporters in the *App*$^{NL-G-F}$ knock-in mice.**

Short running title: **Reduced astrocyte function in *App*$^{NL-G-F}$ mice.**

Authors: Ipsit Srivastava[a], Julen Goikolea[a], Tamer Ayberk Kaya[a], María Latorre-Leal[a], Francesca Eroli[a], Marta Pereira Iglesias[a], Laura Álvarez-Jiménez[a], Luis Enrique Arroyo-García[a], Makoto Shimozawa[a], Per Nilsson[a], André Fisahn[a], Maria Lindskog[a], Silvia Maioli[a,*] and Raúl Loera-Valencia[a,b*].

Affiliation: [a]*Division of Neurogeriatrics; Center for Alzheimer Research; Dept. of Neurobiology, Care Sciences and Society; Karolinska Institutet, 17164 Solna, Sweden*

[b]*Tecnologico de Monterrey. School of Medicine and Health Sciences. Campus Chihuahua, Av. H. Colegio Militar 4700, Nombre de Dios, 31150 Chihuahua, Chih. Mexico.*

[*]These authors contributed equally. Correspondance to: raul.loera@tec.mx and silvia.maioli@ki.se

Supplementary Information.

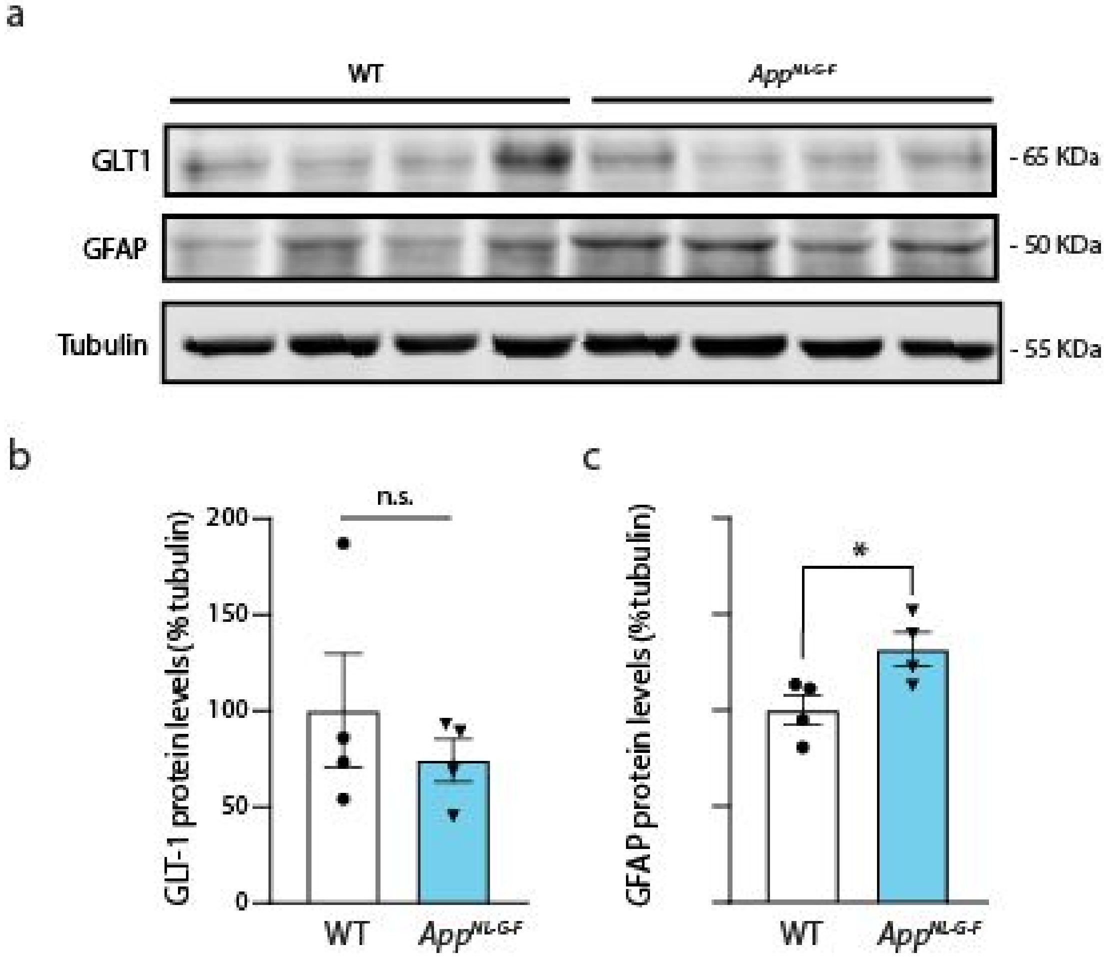

Figure S1. *NL-G-F FACS isolated astrocytes show increased GFAP levels but normal GLT-1 levels.* (a) Western blot analysis of FACS-isolated hippocampal astrocytes from *App*$^{NL\text{-}G\text{-}F}$ and WT mice. (b) Densitometric analysis of synaptosome enriched fractions shows that GLT-1 protein levels do not change significantly in *App*$^{NL\text{-}G\text{-}F}$ compared to WT mice (*n*=4 animals per group; *n.s.*=0.44). (c) Densitometric analysis of GFAP protein levels in FACS-isolated hippocampal astrocytes from *App*$^{NL\text{-}G\text{-}F}$ and WT mice. (*n*=4 animals per group; **p*=0.03).

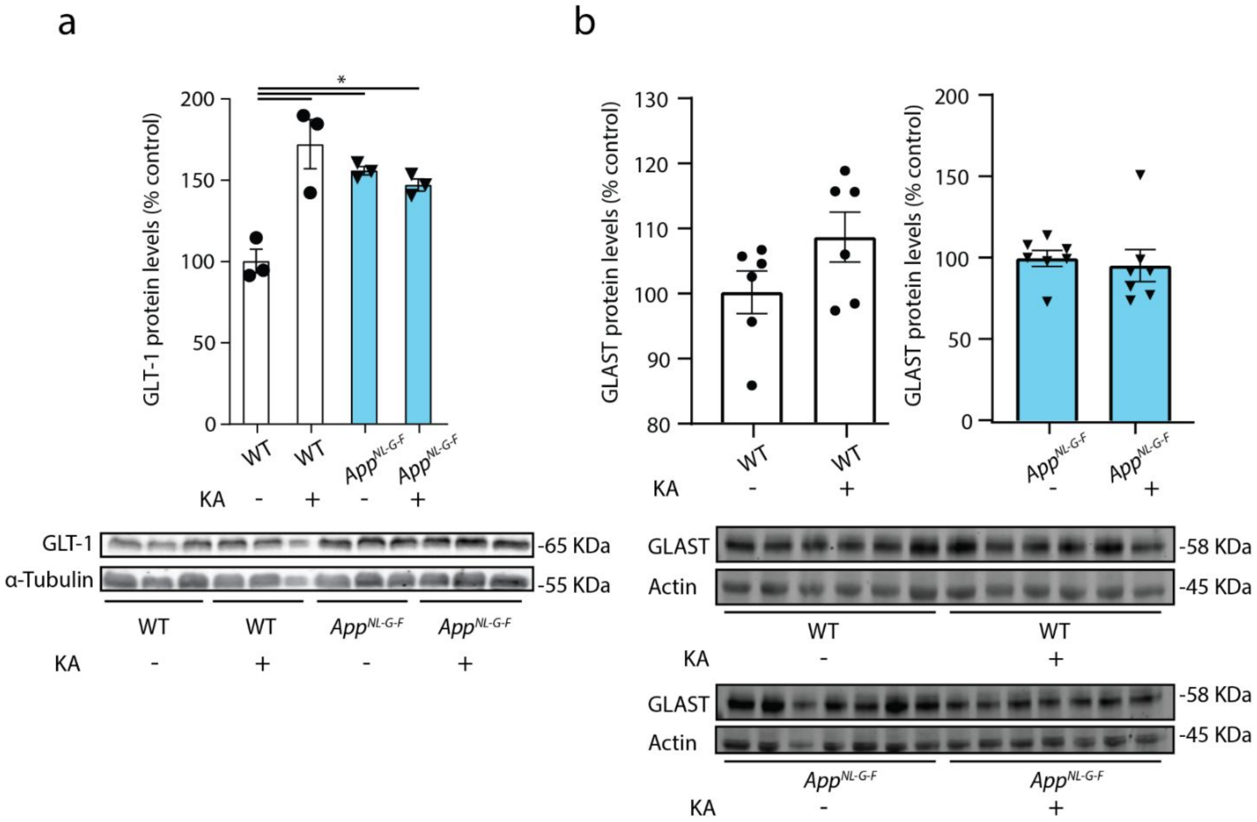

Figure S2: *GLT-1 is upregulated in response to KA in WT, but not App*$^{NL\text{-}G\text{-}F}$ *mice, with no change in GLAST.* Western blots showing the response of thick brain slices to the application of KA to brain slices for 1h compared to similarly treated control slices from *App*$^{NL\text{-}G\text{-}F}$ and WT mice. a) GLT-1 protein levels increase in response to KA in WT animals, while *App*$^{NL\text{-}G\text{-}F}$ GLT-1 levels are elevated in control conditions compared to control WT slices, and do not change with KA stimulation (n=3 animals per group; One-way ANOVA with Sidak´s multiple comparisons, *$p < 0.05$). b) Protein levels of GLAST in WT animals in total protein homogenates from mock-treated slices (KA- , n =6 animals) vs. stimulated slices (KA+, n=6 animals); and in *App*$^{NL\text{-}G\text{-}F}$ mice (KA-, n=7 animals; KA+, n=7 animals). Bars show mean ± SEM.

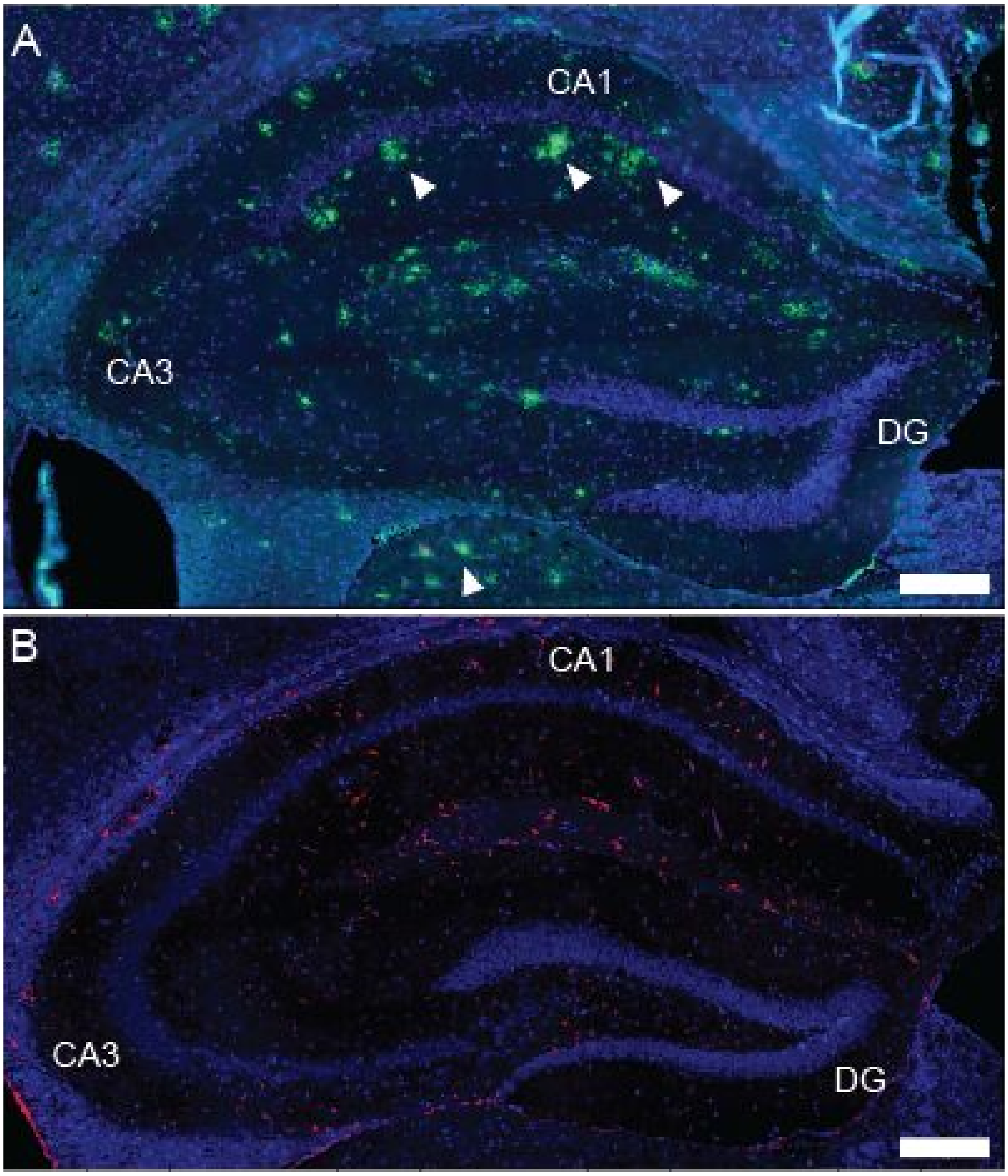

Fig. S3. Representative images from *APP*$^{N\text{-}L\text{-}GF}$ mouse brains used in this study. A) Shows immunoreactivity to amyloid beta in green with aggregates (arrows) throughout the hippocampal regions. B) Shows immunoreactivity to GFAP in red. Both images have output levels increased to improve visualization. Scale bar: 200 µm.

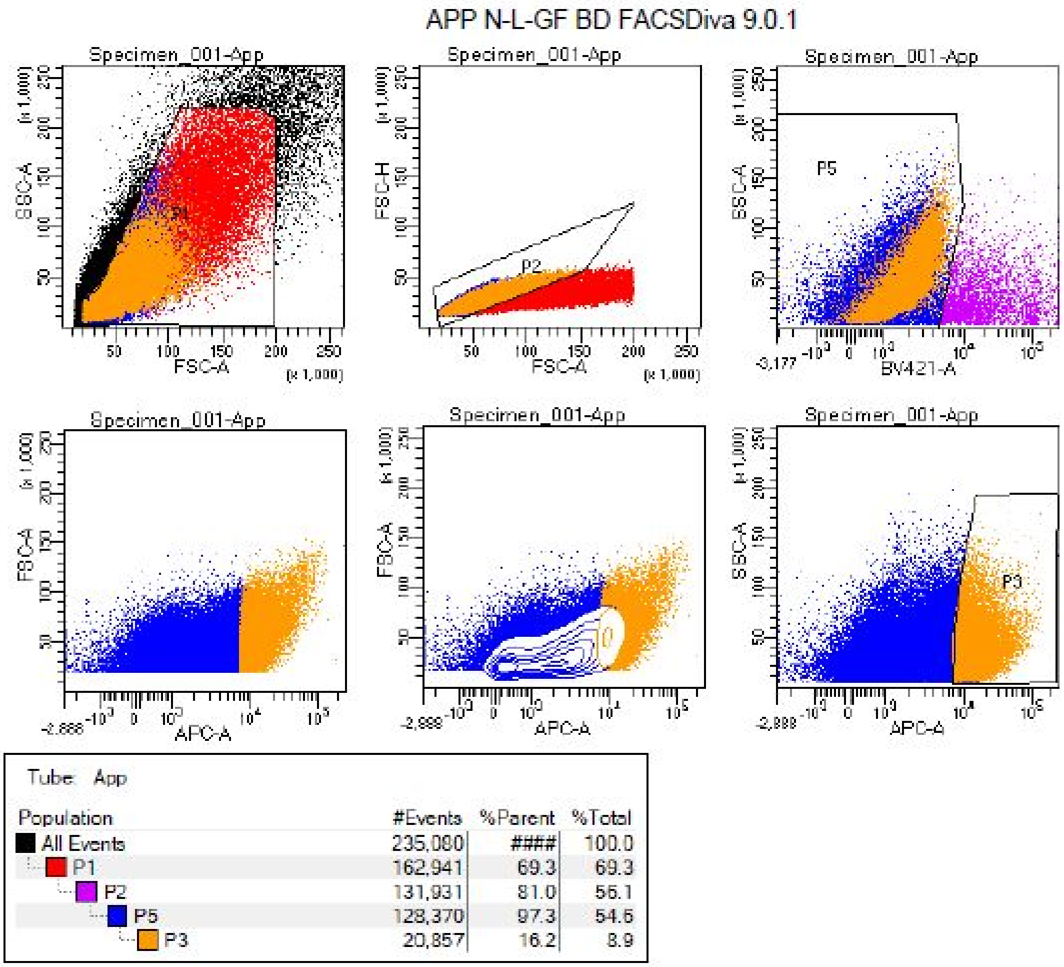

Population	#Events	%Parent	%Total
All Events	235,080	####	100.0
P1	162,941	69.3	69.3
P2	131,931	81.0	56.1
P5	128,370	97.3	54.6
P3	20,857	16.2	8.9

Figure S4. *APP*$^{N\text{-}L\text{-}GF}$ FACS sorting of astrocytes. FACSAria™ III Cell Sorter (BD Biosciences) output report of sorting from 6-month-old APP$^{NL\text{-}G\text{-}F}$ mice.

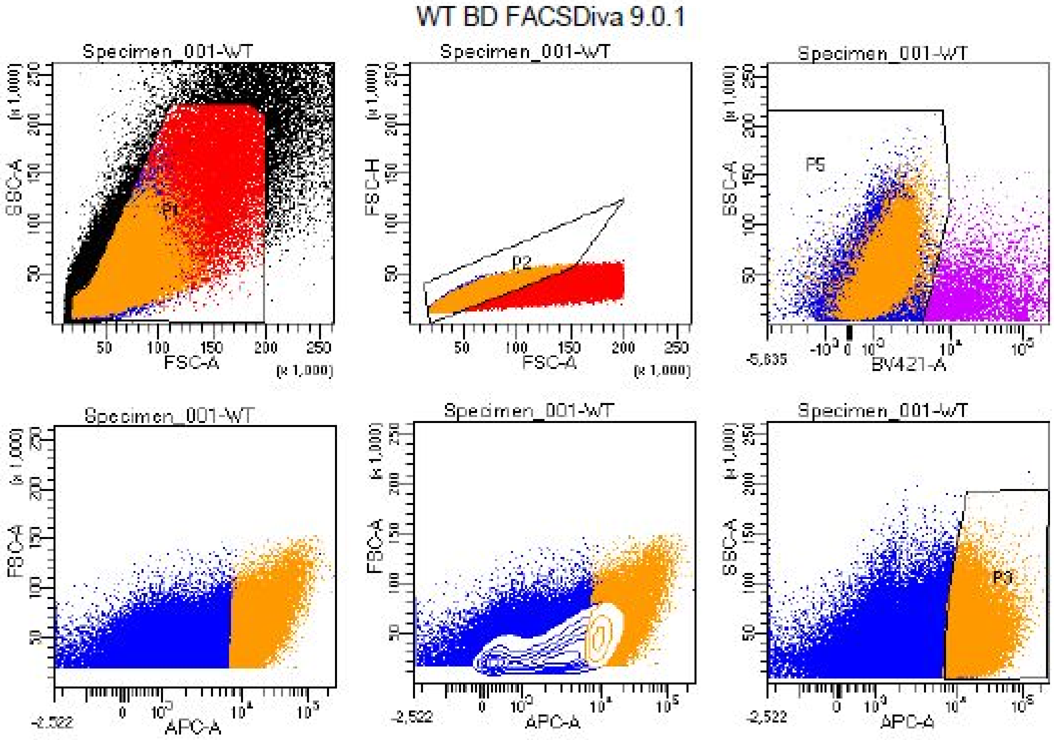

Tube: WT

Population	#Events	%Parent	%Total
All Events	518,561	####	100.0
P1	365,060	70.4	70.4
P2	296,396	81.2	57.2
P5	290,055	97.9	55.9
P3	68,208	23.5	13.2

Figure S5. WT FACS Sorting of astrocytes. FACSAria™ III Cell Sorter (BD Biosciences) output report of sorting from 6-month-old WT mice.
